# Clinical significance of serum soluble B7-H3 in patients with osteosarcoma

**DOI:** 10.1186/s12935-018-0614-z

**Published:** 2018-08-13

**Authors:** Ling Wang, Fu-biao Kang, Guo-chuan Zhang, Juan Wang, Ming-fang Xie, Ying-ze Zhang

**Affiliations:** 1 Department of Orthopedic Oncology, The Third Hospital of Hebei Medical University, Shijiazhuang, Hebei People’s Republic of China; 2 Department of Orthopedic Research Center, The Third Hospital of Hebei Medical University, Shijiazhuang, Hebei People’s Republic of China; 3 Department of Liver Diseases, Bethune International Peace Hospital, Shijiazhuang, Hebei People’s Republic of China; 4 Department of Orthopedic Surgery, The Third Hospital of Hebei Medical University, Shijiazhuang, Hebei People’s Republic of China

**Keywords:** Osteosarcoma, B7-H3, Tumor biomarker, Bone tumor, Prognosis

## Abstract

**Background:**

Increasing data has indicated an association between increased soluble B7-H3 (sB7-H3) levels and unfavorable prognosis in patients with malignancies. However, the level of sB7-H3 and its clinical significance in osteosarcoma (OS) are not well known. In this present study, we investigated whether sB7-H3 levels in serum could be as a tool for differential diagnosis of OS patients.

**Methods:**

Peripheral blood samples from healthy controls, benign bone tumors, and OS patients were collected. Levels of sB7-H3 in serum samples were measured by enzyme-linked immunosorbent assays. The correlation between OS-derived sB7-H3 and clinical features was analyzed, and prognostic significance of the sB7-H3 concentrations and tumor expressions of the biomarkers was then evaluated.

**Results:**

sB7-H3 concentrations were significantly increased in patients with OS than in osteochondroma patients, bone fibrous dysplasia patients and healthy people (*p *< 0.05, respectively). Using 60.94 ng/mL as a cutoff value, the sensitivity and specificity of sB7-H3 was to differentiate between OS patients and other bone benign tumor patients were 75.7% and 83.8%, respectively. In addition, upregulated serum sB7-H3 in patients with OS associated with tumor differentiation, tumor stage, and metastasis status (*p *< 0.05, respectively). The area under the curve value for sB7-H3 (0.868) was markedly higher than those for ALP (0.713) and CA125 (0.789) for differentiating between OS patients and other begin bone tumor patients.

**Conclusions:**

We demonstrated that enhanced sB7-H3 levels correlated with the clinical characteristics of OS patients, and B7-H3 might be a potential biomarker and associated with the pathogenesis of OS.

## Background

Osteosarcoma (OS) is a highly aggressive and life threatening type of malignant bone tumor in children and adolescents [1, 2]. It arises from mesenchymal cells and often offends the metaphyseal region of long bones, including the femur, tibia, or humerus [3]. Due to the considerable improvements in surgery adjuvant chemotherapy, the 5-year survival rate of patients with OS has dramatically increased from approximately 30 to 70% [4]. However, overall survival and prognosis for OS patients was far from satisfactory, because this tumor normally develops and progresses quickly and easily generates drug-resistance after chemotherapy. Additionally, approximately 25% of the patients have already been found distant metastasis when diagnosed, most of these cases are inclined to have poor prognosis [5, 6].

Since OS patients normally present no obvious or specific clinical symptoms, early diagnosis is reliant on the traditional imaging methods, such as X-ray, computed tomography (CT), positron emission tomography (PET)-CT, magnetic resonance imaging (MRI), and scintigraphy [7, 8]. Although biomarkers are increasingly used in cancer treatment to refine risk stratification and augment current clinical decision making tools, there is still absent suitable and useful biomarkers for detecting OS in clinic [9, 10]. For now, alkaline phosphatase (ALP), has been widely applied in clinic and known as a standard tumor biomarker of osteosarcoma, however, sometimes it provides false positives since ALP would be elevated in children after bone damage [11, 12]. Other tumor markers, such as CA125 and CA199, were auxiliary evaluated the status of OS patients, however, these levels in the peripheral blood of OS patients are increased to varying degrees [13, 14]. Therefore, identification of clinically relevant biomarkers for prediction and prognosis is therefore urgently needed for these OS patients.

B7-H3, a recent recognized member of the B7 family, was previously shown an accessory co-stimulator in mediating T cell immune response in cooperation with a putative B7-H3 receptor (B7-H3R) expressed on T cells [15, 16]. However, more recently, Leitner et al. [17] showed that B7-H3 led to a profound downmodulation of T cell responses via inhibition of IFN-γ, IL-2, IL-10, and IL-13 production. Moreover, B7-H3 has been widely detected on the different kinds of tumors and is able to subvert the immune system in immunological and non-immunological way [18–20]. In addition, soluble form B7-H3 (sB7-H3) expression has also been elevated in several tumors and tumor cell lines, and its high levels have been significantly correlated with the invasion and metastasis of tumor [21, 22]. But the precise physiological role of B7-H3 remains debatable. Our previous study showed that B7-H3 was abnormally expressed on the tissue of OS patients and correlated with the development and progression of OS [23, 24]. If soluble form B7-H3 could be validated as a potential biomarker for OS, that would be of great help for the clinical diagnosis. However, whether sB7-H3 can be used as a biological marker for the differential diagnosis of OS and whether sB7-H3 expression correlates with disease progression and prognosis in patients with OS remain unclear.

In the current study, we investigated sB7-H3 levels in serum samples from OS patients and other benign bone tumors using ELISA. In addition, the prognostic significance of sB7-H3 and its correlation with clinical parameters as well were also investigated. We concluded that an enhanced sB7-H3 is correlated with tumor stage and prognosis of OS and might be an innovative non-invasive peripheral blood biomarker for early detection and discrimination of OS.

## Materials and methods

### Patients and plasma samples

Blood samples were collected from 37 OS patients, 42 other benign tumors (including 25 osteochondroma and 17 bone fibrous dysplasia patients, and 40 healthy volunteers at the Third Hospital of Hebei Medical University and Bethune International Peace Hospital between September 2012 and September 2015. All osteosarcoma patients with OS were confirmed by pathology according to the 2002 World Health Organization (WHO) classification standards. The staging of the disease was determined according to the Enneking staging standard, as follows: stage I (low grade malignancy), stage II (high grade malignancy) and stage III (metastasis) [25, 26]. Patients didn’t receive any treatment such as hormones, Chinese traditional drugs, or radio, chemotherapy prior to surgery. Additional inclusion and exclusion criteria were set according to the standards outlined by the WHO. All procedures were approved by and performed in accordance with our institutional review board. Written informed consent was obtained from all patients or their families.

Serum samples were collected before any treatments were initiated and within 24 h after hospitalization and then kept at room temperature for 30 min. Serum was separated by density gradient centrifugation of 5 mL peripheral blood at 3000 rpm for 10 min, and was subsequently aliquot and stored at − 80 °C until use.

### Measure the level of sB7-H3 via ELISA

sB7-H3 concentrations were determined using aB7-H3 ELISA kit (R&D Systems) according to the manufacturer’s instructions. In brief, the serum samples were centrifuged and then transferred to a 96-well plate for incubating 2 h. Then, 200 μL of B7-H3 conjugate was added and incubated for another 2 h at room after washing the plate four times ahead. Next, the same aliquant of substrate solution was added into each well and incubated for 30 min in the dark. Finally, 50 μL stop solution was added to each well, and the absorbance was measured at 450/540 nm.

### Statistical analysis

Results are recorded as mean ± SD. All the experimental data were analyzed by the SPSS 20.0 statistical software package. One-way ANOVA was applied to identify statistical differences between sB7-H3 in OS patients with other benign bone disease or normal patients. Correlation analyses of clinicopathological factors were tested with Pearson Chi square test or Spearman rho test. Receiver operating characteristic (ROC) curves were used to compare the diagnostic accuracy of sB7-H3 in discriminating between OS patients and OS patients with lung metastasis. Survival periods were counted in months from the date of first visit to date of death or last follow-up before study closure. We used Kaplan–Meier method to estimate the overall survival for low and high levels of sB7-H3 expression. Univariate analyses were performed to compare the diagnostic performance of serum B7-H3 and other tumor markers in differentiating between the two groups of patients. Univariate and multivariate analyses (Cox regression models) were used to identify prognostic factors in OS patients. Covariates with *p* values < 0.05 in univariate analysis were included in multivariate analysis. A *p* value < 0.05 was considered as statistically significant.

## Results

### Different expression level of sB7-H3 in OS patients, benign bone diseases and healthy volunteers

**Fig. 1:**
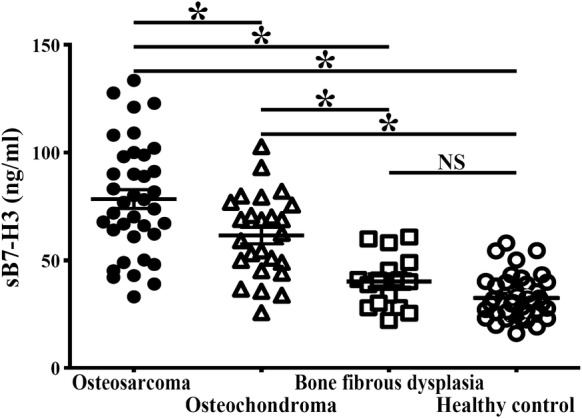
Detection of the different sB7-H3 levels in OS patients, Bone fibrous dysplasia patients, osteochondroma patients and healthy control people (*p < 0.05)

ELISA assay results showed that serum soluble B7-H3 was higher in patients with OS than those with benign tumors or control group. The relative clinical characteristics of these enrolled patients are shown in Table 1. As shown in Fig. 1, the mean concentration of sB7-H3 in the serum of the osteochondroma patients (58.11 ± 9.12 ng/mL, with a range of 25.79–82.09 ng/mL) and bone fibrous dysplasia patients (41.85 ± 10.88 ng/mL, with a range of 22.15–69.08 ng/mL), were comparatively higher than that in the healthy volunteers (32.59 ± 9.02 ng/mL, with a range of 11.18–51.09 ng/mL). In addition, the mean concentration of sB7-H3 in the serum of the OS patients (78.47 ± 21.01 ng/mL, with a range of 33.09–133.56 ng/mL) was significantly higher than that in the osteochondroma patients (*p *< 0.05), bone fibrous dysplasia patients (*p *< 0.001) or healthy volunteers (*p *< 0.001).

**Table 1:** Demographic and clinical data of the study patients’ cohort of OS and non-OS patients

Variable	OS patients	Non-OS patients		*p* value
		Osteochondroma	Bone fibrous dysplasia	
Cases	37	25	17	
Age				
≤ 10	16	9	10	NS
> 10	21	16	7	
Gender				
Male	20	18	10	NS
Female	17	7	7	
Site				
Femur	18	12	7	NS
Tibia	14	8	6	NS
Others	5	5	4	NS
Differentiation status				
High	20	25	17	NS
Low	17	0	0	
Lung metastasis				
Yes	13	0	0	*0.001*
No	24	25	17	
ALP (ng/mL)	108.91 ± 23.02	91.28 ± 12.67	90.34 ± 19.15	*0.031*
CA125 (U/mL)	56.19 ± 14.18	49.21 ± 3.56	39.65 ± 3.44	*0.046*

The italic values show the significance of *p* < 0.05

*NS* not significant

### Diagnostic value of serum sB7-H3 in patients with OS

**Fig. 2:**
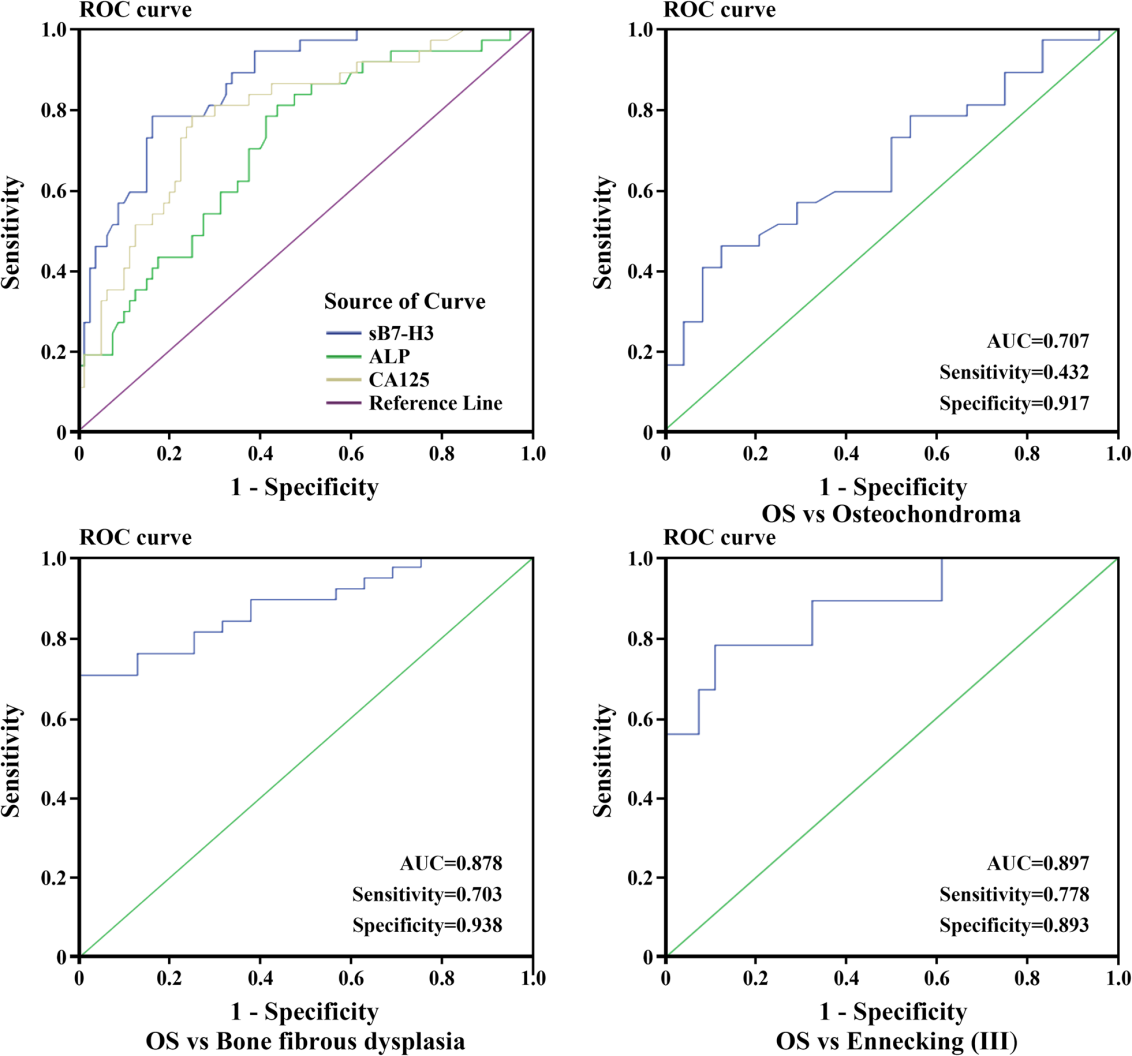
**a** Compared the diagnostic value of sB7-H3 level, ALP and CA125 for distinguishing OS, respectively (*p < 0.05). ROC analysis for sB7-H3 for distinguishing OS patients from those with osteochondroma (**b**). Bone fibrous dysplasia (**c**) or OS patients with III stages from those with II stages (**d**) (*p < 0.05, respectively)

To evaluate the value of sB7-H3 as a serum marker for bone tumor diseases, ROC curve analyses were employed to assess the value of sB7-H3 in differentiating between OS patients, begin bone tumors patients and healthy people. Results showed that the areas under the ROC (AUV) curves reached 0.863 (95% confidence interval = 0.803–0.933). Using a cutoff value (60.94 ng/mL) determined by the Youden Index [27], the sensitivity and specificity values for identifying a patient with osteosarcoma were 75.7% and 83.8%, respectively. To compare the sensitivity and specificity of sB7-H3 with those of other tumor markers for differentiating between OS patients, the diagnostic values of two conventional biomarkers for osteosarcoma, ALP and CA125, were also analyzed. The AUC value of sB7-H3 level was much higher than that of ALP and CA125, (0.713, 95% confidence interval = 0.615–0.811; 0.789, 95% confidence interval = 0.700–0.877; Fig. 2a).

ROC curve analysis result showed that sB7-H3 level was a potential biomarker for distinguishing OS patients from those with osteochondroma patients (AUC of 0.707; sensitivity 0.432; specificity 0.917, Fig. 2b), from those with bone fibrous dysplasia patients (AUC of 0.878; sensitivity 0.703; specificity 0.938, Fig. 2c). To assess the potential of B7-H3 as a diagnostic biomarker to distinguish advanced Ennecking stage OS patients (III) from early clinical stages (II), we got an AUC of 0.897 (95% CI 0.736–0.987) with a sensitivity of 0.778 and a specificity of 0.893 (Fig. 2d). These results demonstrate that serum levels of B7-H3 might be a valuable tumor marker to help partially differentiating patients with osteosarcoma from those with non-osteosarcoma and healthy individuals.

### Correlations between the serum B7-H3 level and clinicopathological features in OS patients

**Fig. 3:**
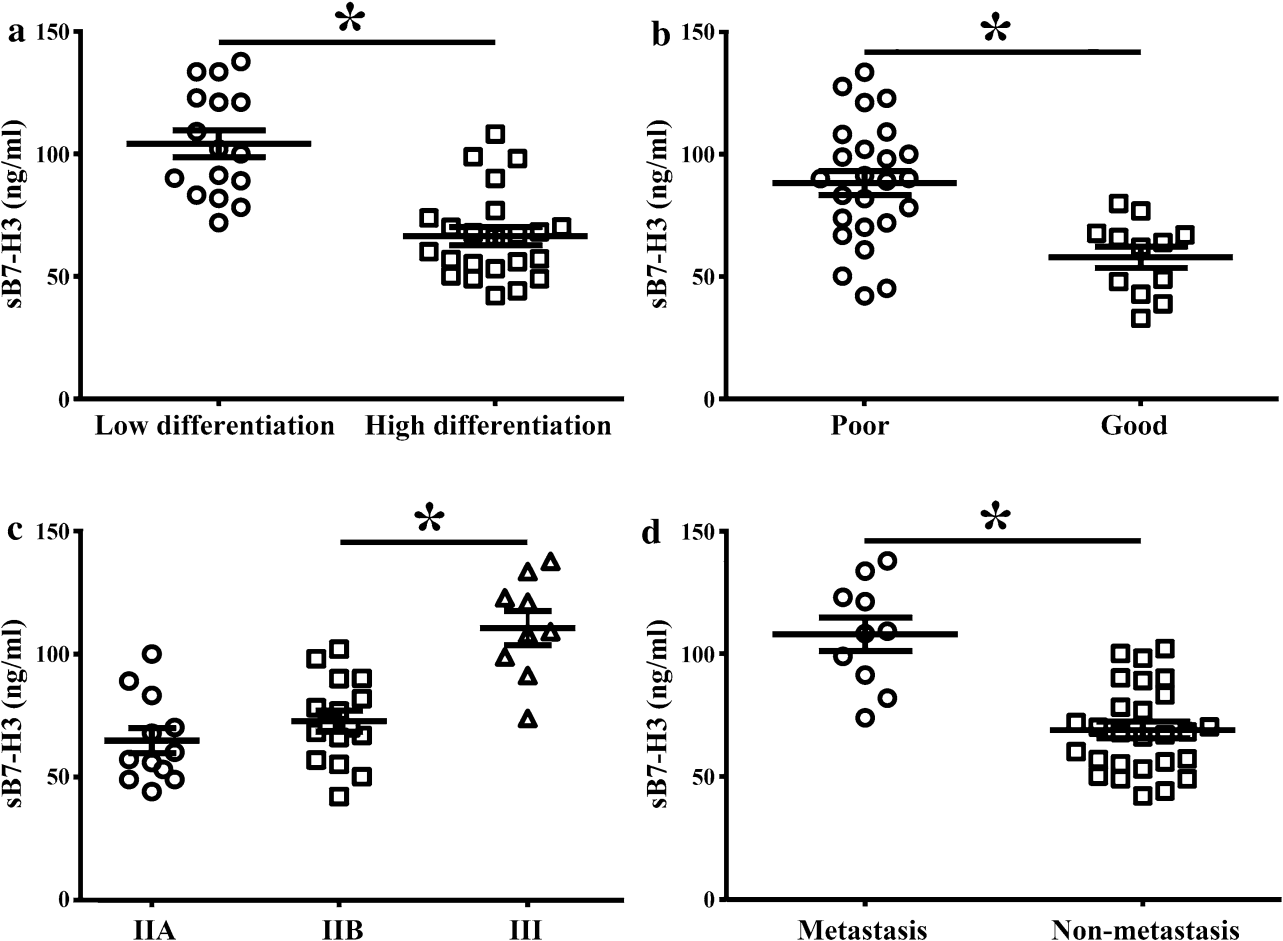
Correlation analysis serum B7-H3 level and OS patients characteristics (**a**) tumor differentiation (**b**) response to chemotherapy (**c**) tumor Ennecking stage (**d**) with and without lung metastases (*p < 0.05, respectively)

Our study showed that serum B7-H3 level was not statistically significant correlated with age (*p *= 0.103), gender (*p *= 0.135), disease site (*p *= 0.801), tumor histology type (*p *= 0.479) and tumor size (*p *= 0.055) in OS patients (Table 2). However, OS patients with low differentiation tumors had greater serum soluble B7-H3 level than those with high differentiated tumors (109.83 ± 18.76 vs 69.84 ± 24.25, *p *= 0.0412, Fig. 3a). Patients with poor response to chemotherapy had relatively higher concentration of sB7-H3 compared to patients with good response (88.27 ± 11.34 vs 58.07 ± 9.98, *p *< 0.05, Fig. 3b). Moreover, levels of sB7-H3 in OS patients tended to increase with increased tumor clinical stages, that is sB7-H3 level of stage III was noticeably higher than that of stage IIB or IIA (110.73 ± 13.87 vs 72.83 ± 9.14 or 64.91 ± 7.43; both *p *< 0.05, Fig. 3c). Likewise, sB7-H3 level of OS patients with distant metastases was remarkably higher than those without metastases (105.8 ± 17.89 vs 80.95 ± 30.12, *p *< 0.05, Fig. 3d). Above all, the results suggest that sB7-H3 level may be closely related to the progression and development of OS.

**Table 2:** Correlations between sB7-H3 level and clinicopathological characteristics

Characteristics	Total number	sB7-H3		*p* value
		High	Low	
Age				
≤ 10	19	14	4	0.395
> 10	18	13	6	
Gender				
Male	22	16	6	0635
Female	15	11	4	
Site				
Femur	24	18	6	0.887
Tibia	9	6	3	
Others	4	3	1	
Size (cm)				
≥ 5	21	15	6	0.555
< 5	16	12	4	
Ennecking stage				
I	12	6	6	*0.037*
II	16	12	4	
III	9	9	0	
Histologic type				
Osteoblastic	27	18	9	0.343
Chondroblastic	8	7	1	
Others	2	2	0	
Differentiation status				
High	23	13	10	*0.003*
Low	14	13	1	
Pulmonary metastasis				
Yes	10	9	1	*0.024*
No	27	17	10	
Response to chemotherapy				
Good	17	9	8	*0.015*
Poor	20	18	2	

The italic values show the significance of *p* < 0.05

### Association of sB7-H3 level with overall survival and prognosis in OS patients

**Fig. 4:**
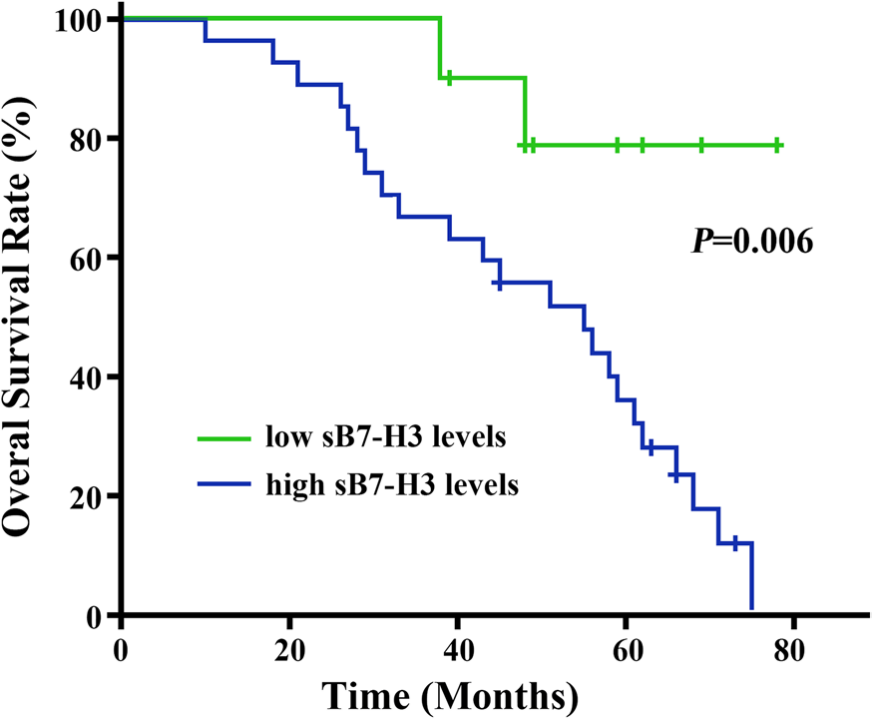
Kaplan–Meier survival curves of all OS patients in relation to sB7-H3 level (*p < 0.05)

The consequences of overall survival for all the 37 detected OS patients analyzed by Kaplan–Meier survival were shown in Fig. 4. It was found that the median survival time for the OS patients with high- and low-sB7-H3 levels were 48.69 and 70.62 months, respectively (Table 3). Patients with lower levels of serum soluble B7-H3 level tended to have longer overall survival in comparison with those expressing higher levels of B7-H3. Cox univariate and multivariate regression analysis were used to determine whether B7-H3 could serve as a reliable predictor for OS prognosis. Univariate analysis indicated that the crucial indexes impacting survival status of OS patients were tumor size, tumor differentiation, TNM stage, metastasis, response to chemotherapy and sB7-H3 level respectively (Table 3). In addition, multivariate Cox regression analysis showed that the worse prognosis in the patients from the high-sB7-H3 group was tendency to be significantly higher than that of the patients from the low-sB7-H3 group (*p *= 0.015). These data demonstrated that increased sB7-H3 level could be functioned as a potential biomarker for predicting poor prognosis in OS patients.

**Table 3:** Univariable and multivariable analysis for of OS disease-free survival

Characteristics	Univariate		Multivariate	
	HR (95% CI)	*p* value	HR (95% CI)	*p* value
Tumor size	2.640 (1.164–5.990)	*0.020*	0.393 (0.623–5.280)	0.274
Tumor differentiation	3.430 (1.515–7.767)	*0.003*	1.232 (0.363–4.181)	0.738
TNM stage	1978 (1.176–3.324)	*0.010*	2.431 (0.779–7.586)	0.056
Metastasis	0.467 (0.203–1.074)	*0.043*	11.416 (1.676–77.76)	*0.013*
Response to chemotherapy	4.497 (1.756–11.516)	*0.002*	2.449 (0.662–9.065)	*0.171*
B7-H3	0.164 (0.038–0.706)	*0.025*	1.042 (1.008–9.065)	*0.015*

The italic values show the significance of *p* < 0.05

## Discussion

Initially, we found that membrane expression of B7-H3 was present in 91.8% of the osteosarcoma lesions, and the intensity of B7-H3 expression in osteosarcoma was significantly correlated with tumor progression, immune cells infiltration and overall survival status, suggesting that B7-H3 could be as a potential predictor and therapeutic target in OS patients [23]. In our following study, we showed that B7-H3 was bond to and mediated by onco-miRNA miR-124 in cell growth, invasion and migration [24]. Determining how to increase the rate of OS detection and improve early identification of benign and malignant bone tumor is very important. Inspired by previous studies, our current research mainly focuses on using soluble form of B7-H3 to predict the prognosis of osteosarcoma. Herein, we measured sB7-H3 expression in serum samples obtained from OS patients, benign bone tumor and healthy people using sandwich ELISA. Results showed that sB7-H3 level in OS patients was significantly higher than that in benign bone tumor patients, and sB7-H3 level in benign bone tumor patients was comparatively higher than that in healthy people. Therefore, it is presumed that serum sB7-H3 may serve as a potential biomarker for OS patients.

Osteosarcoma is characterized by production of osteoid tissue or immature bone tissue, therefore, biochemical markers reflexing bone turnover and tumor indicator are normally considered clinically useful to predict OS development and progress [10, 28, 29]. To further determine the value of circulating sB7-H3 as a diagnostic marker, we assessed it in parallel with two other tumor markers, including ALP and CA125 in patients with OS. It has been reported that sB7-H3 level can predict the presence of non-small-cell lung cancer, hepatocellular carcinoma in compared to other tumor markers tested including CA125, CA153, CA199, and CEA [30]. In our study, when the sB7-H3 level cutoff as a marker for OS was 60.94 ng/mL, the predicted values had a sensitivity of 75.7% and specificity of 83.8%. sB7-H3 was a promising diagnostic biomarker to distinguish OS from benign bone tumors and healthy controls, and can also distinguish advanced from early OS stages. Compared with the other two biomarkers, the sensitivity of sB7-H3 was almost equal to that of ALP and CA125, but the specificity was much higher than those of two biomarkers, suggesting that serum sB7-H3 may be a better tumor marker than the other options for differentiating between patients with and without OS. During recent years, strong efforts were directed to establish non-invasive biomarkers from liquid biopsies. The clinical relevance of various newly established blood-based biomarkers comprising circulating tumor cells (CTCs), circulating cell-free nucleic acids or tumor-educated platelets is being tested in cancer patients. If sB7-H3 is an indicative biomarker for predicting OS progression, trace of little sB7-H3 changes in CTCs or exosomes would be helpful to monitor tumor micrometastasis and even might be amenable for OS immunotherapy in the future study under application of digital PCR approaches or next generation sequencing (NGS). Based on our results, we reason that the observed correlation trend of high B7-H3 circulating levels with tumor size, tumor differentiation, TNM stage, and metastasis could be biologically significantly relevant. The present study revealed a link between sB7-H3 level and overall survival in patients with OS. Moreover, univariate and multivariable analysis validated that sB7-H3 is an independent and significant factor influencing postsurgical survival time in patients with OS. These results indicate that measuring a circulating B7-H3 levels shows favorable sensitivity and specificity which could be detected by protein expression analysis of patient’s serum rather than the simple focus on dysregulated mRNAs in tumor cells or tissues, sB7-H3 might be easier to obtain and provide application into the prediction and progress for OS patients. Patients with high serum B7-H3 expression may be at high risk for OS progression and therefore may benefit from more aggressive treatment or surveillance. By contrast, patients with OS that exhibit low-sB7-H3 expression might be better suited for less intensive management. Based our above results, B7-H3 blockade may offer a feasible tumor suppression function by reinvigorating antitumour immune response. Targeting B7-H3 may be valuable for the development of a new strategy for antitumor therapy in OS. However, this study also has several limitations. The research cohort is comparatively small, and most cases belong to the type of conventional osteosarcoma since OS is a rare kind of tumor. Bedsides, the applicability of our results to inoperable advanced primary OS requires further study.

## Conclusions

The serum level of sB7-H3 reflected tumor progression in patients with OS, and serum sB7-H3 levels were significantly correlated with the clinical stage and patients’ prognosis. These results suggest that serum sB7-H3 levels can be functioned as a potential biomarker for predicting poor prognosis in OS patients and might be partially helpful in differential diagnosis of OS and other tumors.

## Abbreviations

sB7-H3: soluble B7-H3
OS: osteosarcoma
ELISA: enzyme-linked immunosorbent assays
AUC: area under the curve
ALP: alkaline phosphatase
CT: computed tomography
B7-H3R: B7-H3 receptor
WHO: World Health Organization
ROC: receiver operating characteristic

## References

[1] Valery PC, Laversanne M, Bray F (2015). Bone cancer incidence by morphological subtype: a global assessment. Cancer Causes Control.

[2] Anfinsen KP, Devesa SS, Bray F, Troisi R, Jonasdottir TJ, Bruland OS, Grotmol T (2011). Age-period-cohort analysis of primary bone cancer incidence rates in the United States (1976–2005). Cancer Epidemiol Biomarkers Prevent.

[3] Kansara M, Teng MW, Smyth MJ, Thomas DM (2014). Translational biology of osteosarcoma. Nat Rev Cancer.

[4] Hasei J, Sasaki T, Tazawa H, Osaki S, Yamakawa Y, Kunisada T, Yoshida A, Hashimoto Y, Onishi T, Uno F (2013). Dual programmed cell death pathways induced by p53 transactivation overcome resistance to oncolytic adenovirus in human osteosarcoma cells. Mol Cancer Ther.

[5] Savage SA, Mirabello L (2017). Bone cancer: is the osteosarcoma genome targetable?. Nat Rev Endocrinol.

[6] Hutanu D, Popescu R, Stefanescu H, Pirtea L, Candea A, Sarau C, Boruga O, Mehdi L, Ciuca I, Tanasescu S (2017). The molecular genetic expression as a novel biomarker in the evaluation and monitoring of patients with osteosarcoma-subtype bone cancer disease. Biochem Genet.

[7] Lindsey BA, Markel JE, Kleinerman ES (2017). Osteosarcoma overview. Rheumatol Ther.

[8] Harrison DJ, Parisi MT, Shulkin BL (2017). The role of 18F-FDG-PET/CT in pediatric sarcoma. Semin Nucl Med.

[9] Ram Kumar RM, Boro A, Fuchs B (2016). Involvement and clinical aspects of microRNA in osteosarcoma. Int J Mol Sci.

[10] Bernardini G, Laschi M, Geminiani M, Santucci A (2014). Proteomics of osteosarcoma. Expert Rev Proteomics.

[11] Kim SH, Shin KH, Moon SH, Jang J, Kim HS, Suh JS, Yang WI (2017). Reassessment of alkaline phosphatase as serum tumor marker with high specificity in osteosarcoma. Cancer Med.

[12] Pujari-Palmer M, Pujari-Palmer S, Lu X, Lind T, Melhus H, Engstrand T, Karlsson-Ott M, Engqvist H (2016). Pyrophosphate stimulates differentiation, matrix gene expression and alkaline phosphatase activity in osteoblasts. PLoS ONE.

[13] Ma J, Liang L, Gu B, Zhang H, Wen W, Liu H (2013). A retrospective study on craniofacial fibrous dysplasia: preoperative serum alkaline phosphatase as a prognostic marker?. J Craniomaxillofac Surg.

[14] Wu J, Guo A, Li Q, Wang D (2017). Meta-analysis of clinical significance of p53 protein expression in patients with osteosarcoma. Future Oncol.

[15] Chapoval AI, Ni J, Lau JS, Wilcox RA, Flies DB, Liu D, Dong H, Sica GL, Zhu G, Tamada K (2001). B7-H3: a costimulatory molecule for T cell activation and IFN-gamma production. Nat Immunol.

[16] Sun M, Richards S, Prasad DV, Mai XM, Rudensky A, Dong C (2002). Characterization of mouse and human B7-H3 genes. J Immunol.

[17] Leitner J, Klauser C, Pickl WF, Stockl J, Majdic O, Bardet AF, Kreil DP, Dong C, Yamazaki T, Zlabinger G (2009). B7-H3 is a potent inhibitor of human T-cell activation: no evidence for B7-H3 and TREML2 interaction. Eur J Immunol.

[18] Lee YH, Martin-Orozco N, Zheng P, Li J, Zhang P, Tan H, Park HJ, Jeong M, Chang SH, Kim BS (2017). Inhibition of the B7-H3 immune checkpoint limits tumor growth by enhancing cytotoxic lymphocyte function. Cell Res.

[19] Ni L, Dong C (2017). New B7 family checkpoints in human cancers. Mol Cancer Ther.

[20] Altan M, Pelekanou V, Schalper KA, Toki M, Gaule P, Syrigos K, Herbst RS, Rimm DL (2017). B7-H3 expression in NSCLC and its association with B7-H4, PD-L1 and tumor-infiltrating lymphocytes. Clin Cancer Res.

[21] Zhao L, Xie C, Liu D, Li T, Zhang Y, Wan C (2017). Early detection of hepatocellular carcinoma in patients with hepatocirrhosis by soluble B7-H3. J Gastrointest Surg.

[22] Baral A, Ye HX, Jiang PC, Yao Y, Mao Y (2014). B7-H3 and B7-H1 expression in cerebral spinal fluid and tumor tissue correlates with the malignancy grade of glioma patients. Oncol Lett.

[23] Wang L, Zhang Q, Chen W, Shan B, Ding Y, Zhang G, Cao N, Liu L, Zhang Y (2013). B7-H3 is overexpressed in patients suffering osteosarcoma and associated with tumor aggressiveness and metastasis. PLoS ONE.

[24] Wang L, Kang FB, Sun N, Wang J, Chen W, Li D, Shan BE (2016). The tumor suppressor miR-124 inhibits cell proliferation and invasion by targeting B7-H3 in osteosarcoma. Tumour Biol.

[25] Jo VY, Fletcher CD (2014). WHO classification of soft tissue tumours: an update based on the 2013 (4th) edition. Pathology.

[26] Doyle LA (2014). Sarcoma classification: an update based on the 2013 World Health Organization classification of tumors of soft tissue and bone. Cancer.

[27] Youden WJ (1950). Index for rating diagnostic tests. Cancer.

[28] Byrum S, Montgomery CO, Nicholas RW, Suva LJ (2010). The promise of bone cancer proteomics. Ann N Y Acad Sci.

[29] Davicioni E, Wai DH, Anderson MJ (2008). Diagnostic and prognostic sarcoma signatures. Mol Diagn Ther.

[30] Zhang G, Xu Y, Lu X, Huang H, Zhou Y, Lu B, Zhang X (2009). Diagnosis value of serum B7-H3 expression in non-small cell lung cancer. Lung Cancer.

